# Getting closer to modeling the gut-brain axis using induced pluripotent stem cells

**DOI:** 10.3389/fcell.2023.1146062

**Published:** 2023-03-31

**Authors:** Vanessa Hall, Katja Maria Sahlgren Bendtsen

**Affiliations:** Group of Brain Development and Disease, Department of Veterinary and Animal Sciences, Faculty of Health and Medical Sciences, University of Copenhagen, Copenhagen, Denmark

**Keywords:** gut microbiome, gut barrier, blood-brain barrier, gut-brain axis, induced pluripotent stem cells, organ-on-a-chip, gut-brain-axis-on-a-chip

## Abstract

The gut microbiome (GM), the gut barrier, and the blood-brain barrier (BBB) are key elements of the gut-brain axis (GBA). The advances in organ-on-a-chip and induced pluripotent stem cell (iPSCs) technology might enable more physiological gut-brain-axis-on-a-chip models. The ability to mimic complex physiological functions of the GBA is needed in basic mechanistic research as well as disease research of psychiatric, neurodevelopmental, functional, and neurodegenerative diseases, such as Alzheimer’s disease and Parkinson’s disease. These brain disorders have been associated with GM dysbiosis, which may affect the brain *via* the GBA. Although animal models have paved the way for the breakthroughs and progression in the understanding of the GBA, the fundamental questions of exactly when, how, and why still remain unanswered. The research of the complex GBA have relied on equally complex animal models, but today’s ethical knowledge and responsibilities demand interdisciplinary development of non-animal models to study such systems. In this review we briefly describe the gut barrier and BBB, provide an overview of current cell models, and discuss the use of iPSCs in these GBA elements. We highlight the perspectives of producing GBA chips using iPSCs and the challenges that remain in the field.

## 1 Introduction

Animal studies related to studying disease mechanisms and medical advances are difficult to replace. When using animal models, we have a responsibility to consider animal welfare and ethical concerns and ensure robust and replicable research. Despite the number of animals used in pre-clinical and pharmacology research, there is a surprisingly low percentage of animal research that successfully translates to human clinical trials (–90% of drug candidates fail Phase 1 (Seyhan, 2019)). To counter these issues, there has been a rise in the development of novel *in vitro* methods that reduce the need for animal studies. These new methods are often referred to as non-animal models (NAMs), although this abbreviation is also synonymous with non-animal methods or new approach methodologies, which are also applicable in this context. Increasingly advanced scientific and technical developments make us able to mimic complex physiological functions by combining different cell types and formats. Such models include organoids (Dutta et al., 2017; Lehmann et al., 2019; Kim et al., 2020), multi-well systems (Baudoin et al., 2012; Dutta et al., 2017; Roh et al., 2021), as well as microphysiological organ-on-a-chip (OoC) models (Vernetti et al., 2017; Ronaldson-Bouchard and Vunjak-Novakovic, 2018; Zhang et al., 2018). Organoids are multicellular 3D spheres that differentiate and self-organize to reconstruct the features of cell–cell interactions of specific tissues (Dutta et al., 2017; Liu et al., 2018). The OoC models may use the Transwell® system, consisting of a microporous semi-permeable membrane suspended in culture wells, which permits diffusion and separates vascular and parenchymal compartments, and/or comprise a microfluidic system (Bhatia and Ingber, 2014) connecting one or more compartments in horizontal or vertical alignment. OoCs can model single multi-cellular organ systems using organoids or complex multi-organ systems (human-on-a-chip) (Leung et al., 2022). The different compartments mimic a specific tissue, which can be exposed to compounds, potentially affecting other compartments. Complex tissues or cell types can be modeled with either primary or immortalized cell lines in co-cultures or by induced pluripotent stem cells (iPSCs). One of the challenges in producing good OoC models is recapitulating relevant biology that is physiologically comparable to that found in complex tissues *in vivo*. Human induced pluripotent stem cells (iPSCs) have become a popular cell type to incorporate into OoCs, as the same parental cell line can be used to differentiate into several cell types, thereby recapitulating complex tissues from the same genetic background. The possibilities of better mimicking organs or tissues has increased as a consequence of iPSC technology. IPSCs also have advantages over embryonic stem cells as disease-specific iPSCs can be produced from patient-derived cells or *via* gene editing. More complex iPSC-based differentiation systems are being developed to replicate human tissue-level and organ-level dysfunction, relevant for a range of research areas such as disease modeling, drug screening, and host–pathogen interactions. Of the different organs that can be produced on a chip, the brain and the gut are two organ systems that have become of particular interest, based on the striking and important relationships between gut health and brain function. In order to study the mechanisms that transcend these two organ systems and their inter-compartmental connections, it will be important to determine and recapitulate the key biological elements required for development of such a model system.

**FIGURE 1:**
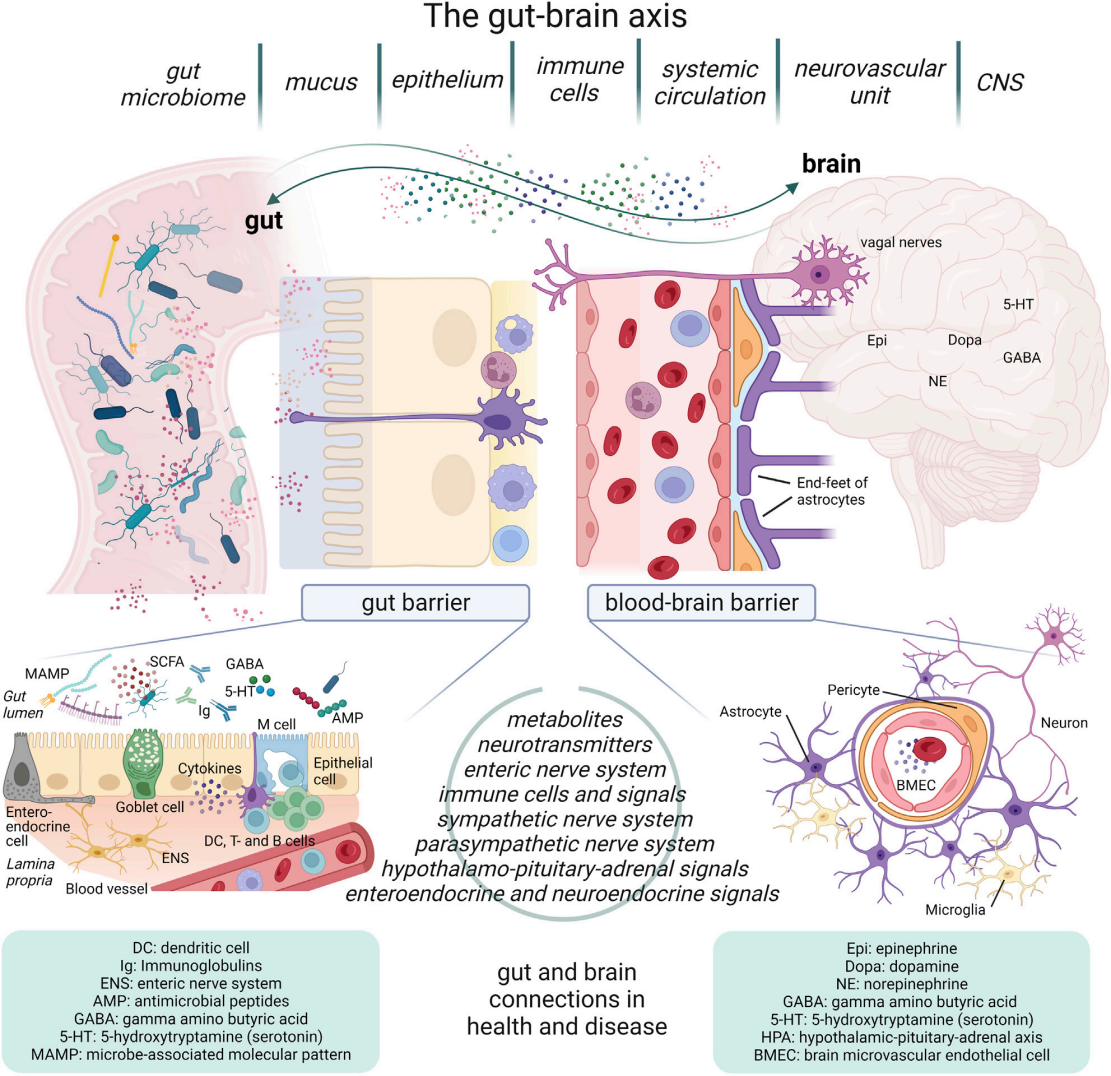
Overview of the gut-brain axis. The gut-brain axis (GBA) constitutes multiple connections between the gut and the brain separated by the gut barrier and the blood-brain barrier (BBB). The gut microbiome contributes significantly with a wide range of molecules, including microbe-associated molecular patterns (MAMPs), immune signaling, metabolites, and neurotransmitters. The enteric nerve system is considered a “second brain”, and it is part of the conduction system leading signals on to the CNS, along with vagal and endocrine pathways from specialized cells of the gut mucosa. The systemic blood system is separated from the brain’s vascular system by the BBB, consisting of the neurovascular unit of brain microvascular endothelial cells, pericytes, and astrocytes. Together these two barriers outline the significant functional and physiological diversity of the GBA, relevant in health and disease. (The figure was created with Biorender).

The gut-brain axis (GBA) links the gut and the brain both anatomically and physiologically (Figure 1). The GBA is considered a bidirectional communication network of neurons and endocrine-, humoral-, metabolic-, and immunological signaling molecules traveling between gut and brain (Cryan et al., 2019). Implied in this definition is a holobiontic view of the body as a host in an ecosystemic symbiosis with the (gut) microbiome to function in immunity, nutrition, and health (Reynoso-García et al., 2022). Even developmental influence of the gut microbiome on the brain is largely supported, particularly from pre-clinical studies, such as colonization-driven maturing of the ENS in germ-free mice (Heijtz et al., 2011; De Vadder et al., 2018; Needham et al., 2020), as well as contributions in aging (Boehme et al., 2023). However, the brain and the gut are two compartments separated by intrinsic barriers, namely the gut barrier and the blood-brain barrier (BBB) (Figure 1).

There is strong epidemiological evidence and an accumulating body of pre-clinical studies that show an unhealthy gut is linked to brain disorders, diseases and mental health, although exact mechanisms are unknown. The GBA has been implicated in a range of neurodevelopmental, functional, and psychiatric disorders including multiple sclerosis, neurodegenerative diseases (Alzheimer’s disease, (AD) and Parkinson’s disease, (PD)), as well as major depressive - and mood disorders (Rutsch et al., 2020; Sorboni et al., 2022). A recently published study investigated the hypothesis that GM is involved in regulation of tau pathology and tau-mediated neurodegeneration in an ApoE isoform–dependent manner relating to AD. The GM of a mouse model of tauopathy (P301S tau transgenic mice) expressing human ApoE isoforms (ApoE3 and ApoE4) was changed by germ-free conditions or short-term antibiotic treatment in early life. At euthanasia at 40 weeks, the GM manipulation had resulted in a marked reduction in tau pathology and neurodegeneration depending on ApoE isoform. Astrocytes and microglia were observed to be in a more homeostatic-like state, indicating that GM significantly influenced neuroinflammation and tau-pathology in this model (Seo et al., 2023). Epidemiological studies show that patients with the above listed brain disorders and diseases, typically carry a dysbiotic gut microbiome often characterized by lack of microbial richness (alpha diversity) and skewness (beta diversity) compared to healthy people. A recent meta-analytical study explored the GM characteristics of 1519 psychiatric patients in comparison to 1429 control participants (Nikolova et al., 2021). A trans-diagnostic pattern was found in patients suffering from major depressive disorder, bipolar disorder, psychosis, schizophrenia, and anxiety. Specific anti-inflammatory butyrate-producing gut bacteria were found to be depleted, and pro-inflammatory bacteria were enriched (Nikolova et al., 2021). In the case of autism, Kang et al. found that the GM composition of autistic children with gastro-intestinal symptoms was characterized by significantly lower abundance of the genera Prevotella, Coprococcus, and unclassified Veillonellaceae, which are carbohydrate-degrading and/or fermenting bacteria (Kang et al., 2013). The phenomenon of phenotype-creating gut microbiota was initially proven as a concept within obesity research, where Turnbaugh and colleagues showed that fecal microbiota transplant (FMT) from obese mice caused weight gain in lean mice and reversely so (Turnbaugh et al., 2006). We know that the GM closely affects energy harvest, obesity, and related disease mechanisms such as diabetes, by regulating metabolic homeostasis. But the hypothesis of our GM shaping our mental health is still controversial. A new study investigated the composition of the fecal microbiome in relation to depressive symptoms in a cohort of 1,054 participants with validation in another cohort of 1,539 subjects. Thirteen microbial taxa were found to be correlated with depressive symptoms (genera Eggerthella, Subdoligranulum, Coprococcus, Sellimonas, Lachnoclostridium, Hungatella, Ruminococcaceae (UCG002, UCG003 and UCG005), Lachnospiraceae UCG001, Eubacterium ventriosum and Ruminococcusgauvreauiigroup, and family Ruminococcaceae). These bacteria synthesize key neurotransmitters involved in depression, namely glutamate, butyrate, serotonin and gamma amino butyric acid (GABA) (Radjabzadeh et al., 2022). A systematic review from 2020 of fecal microbiota transplants in twenty-one clinical, pre-clinical with human donors, and entirely pre-clinical studies, found that all studies reported a decrease in depressive and anxiety-related symptoms and behaviors after transplant of microbiota from healthy donors (Chinna Meyyappan et al., 2020). The reverse was also reported, where transplant of microbiota from psychiatrically ill donors transferred depressive and anxiety-related symptoms and behaviors (Chinna Meyyappan et al., 2020). Together these studies show a huge potential for GM-manipulative treatment of psychiatric and neurological disease, though more consistent results are needed (Vendrik et al., 2020). Moreover, it pinpoints the fact that the GBA is a significant mechanistic system in need of further research.

IPSC technology has progressed the use and applications of *in vitro* models of the gut barrier, the GM, and the blood-brain barrier. In this review we focus on how far the field has progressed in modeling these elements of the GBA. We highlight the perspectives of producing GBA chips using iPSCs and the challenges that remain in the field.

## 2 Modeling the barriers of the gut-brain axis—From cell lines to iPSC technology

### 2.1 The gut barrier

The gut barrier is located between the external milieu of the gut lumen and the internal milieu. It is defined by a combination of physical, biochemical, and immunological components. The gut barrier has the complex task of segregation (internal from external milieu) and surveillance (pathogens and dietary antigens). The gastro-intestinal tract harbors around two kilos of microbiota consisting of bacteria, viruses, fungi, and protozoa that contribute to energy harvest and are essential to metabolism and immune functions. The GM contributes to a barrier of complex cellular diversity and regulation (Allam-Ndoul et al., 2020). The gut barrier segregates the majority of contents that pass through the gut lumen, including familiar or novel dietary components, from the cells and blood system directly beneath, until transported, processed, or evaluated as safe by immune surveillance mechanisms. The gut barrier carries out this task by functioning as a firewall that also safeguards against pathogenic microorganisms (Vancamelbeke and Vermeire, 2017). It is composed of the epithelium consisting of epithelial cells, dendritic cells, systemic immune cells, anti-microbial proteins, and secreted antibodies (Figure 1). Central is the physical barrier of the epithelial cells bound together by tight junctions. The epithelial cells are highly polarized with the apical plasma membrane towards the lumen. The basolateral surfaces are associated with the lamina propria containing immune cells from the circulation. Intestinal epithelial stem cells are located at the bottom of crypts that form between villi. These intestinal stem cells give rise to differentiation and maturing of distinct epithelial cell types (Luo et al., 2022). Goblet cells, enteroendocrine cells, Paneth cells, tuft cells and species-specific cup cells (Gerbe and Jay, 2016) are located in the villi and mature Paneth cells dwell at the crypt bottoms. In addition, phagocytic and transcytotic microfold cells are found within follicle-associated epithelia that overly gut-associated lymphoid tissues (Yu and Gao, 2015). Scattered between the epithelial cells are the mucus-producing goblet cells that vary in type of mucin and number depending on the specific compartment of the gut. The mucin layer is composed of an inner attached layer and an outer loose layer, and commensal bacteria, such as Akkermansia muciniphila that assist in the maintenance of the mucus layer. Mucin degradation and turnover by muciniphilic GM has been found to be particularly important for barrier health and immune functions. In fact, these particular bacteria are often reported as decreased in patients and in animal models of inflammatory diseases (Bendtsen et al., 2015; Paone and Cani, 2020). The conserved innate Toll-like receptors (TLRs) on the epithelium and immune cells of the gut (also present in the brain and in other tissues) are in the forefront of the intricate *defend or tolerate* immune balance (Beutler, 2009), contributing to barrier—and body health. The TLRs are essential innate membrane-bound receptors that each recognize specific bacterial ligands—13 TLRs have been identified so far (humans: TLR1-13, mice: TLR1-9 and 11-13), with some variations between species. Some are more abundant in specific cells than others, and the expression of TLRs has been discovered to be highly compartmentalized along the gastrointestinal tract and differentiated in apical-basolateral locations (Yu and Gao, 2015; Hug et al., 2018). Recognition by TLRs of microbial antigens strengthens the intestinal epithelial barrier function by inducing the tightening of the intercellular junctions, secretion of mucus and antimicrobial peptides, and the production of reactive oxygen species (Burgueño and Abreu, 2020). It is clear that the gut barrier is a multicellular, complex tissue that has immunomodulatory and protective properties that is difficult to replicate in its entirety *in vitro*.

#### 2.1.1 Modeling the healthy gut using iPSCs

Complex gut models have been developed with primary cells from intestinal biopsies, such as the Small Intestine-on-a-Chip (Kasendra et al., 2018). However, isolated primary human- or animal-derived cell lines pose challenges to maintain and have limited proliferation rates. These limitations make immortalized cancer cell lines more attractive substitutes. The human adenocarcinoma-derived colonic epithelial cell line Caco-2 is widely used in studies of gut epithelial barrier functions, including drug transport and toxicity studies (Wang et al., 2023), and is used as a standard reference tool for absorption (Sambuy et al., 2005; Peng et al., 2007). More complex co-cultures have been developed with human colorectal adenocarcinoma-derived HT29-MTX cells, which differentiate to mucus-producing goblet cells (Lesuffleur et al., 1990), and addition of M-cells/lymphocytes mimicking immune functions. Some challenges arise in these triple models, such as loose tight junctions and non-uniform mucus layers (Xu et al., 2021), and it is still being researched which of the current co-culturing methods are most useful for prediction of human gut permeability (Lozoya-Agullo et al., 2017). Sources of intestinal epithelial cells that are physiologically relevant are crucial for drug transport studies of human absorption, but they are also required for complex physiological model systems of the GBA. Human immortalized cell lines, like Caco-2, are easy to culture, maintain, and scale, but they lack complete mimicry of the *in vivo* intestinal phenotypes. However, with the improvement of human iPSC differentiation, the perspectives of approximating *in vivo* conditions have increased significantly. A handful of studies have used iPSCs to produce the intestinal epithelium and evidence suggests these are of equal or superior quality when compared to commonly used cancer (Caco-2) cell lines. In one study, iPSC-derived intestinal cell monolayers cultured with Transwell® exhibited high transepithelial/transendothelial electrical resistance (TEER) with low permeability, indicative of strong tightness of the barrier. The iPSC-derived intestinal cells also exhibited expected marker expression and basic functional monolayer formation, similar to or better than the Caco-2 cell line (Kauffman et al., 2013). Improved cell models have been applied in testing drug absorption rates. One study generated villin- and zonula occludens-1 (ZO1)-positive intestinal epithelial cells from human iPSC-derived intestinal progenitor cells, and found the drug absorption rates of these monolayers to be highly correlated with those in humans, as well as similar in expression levels of the drug metabolism enzyme cytochrome P450 (Takayama et al., 2019). Studies have also demonstrated the potential of iPSCs to differentiate and organize into complex and more functional organ-like structures with motor function, such as the induced gut consisting of a lumen surrounded by epithelium, connective tissue, and musculature. The induced gut (iGut) was developed by a hanging drop culture system, and was able to spontaneously contract and mimic peristalsis with content transportation. The iGut was reported to be composed of the enteric components of the three germ layers: epithelial cells (endoderm), smooth muscle cells (mesoderm), interstitial pacemaker cells (mesoderm), and enteric neurons (ectoderm). This was the first demonstration of the *in vitro* differentiation potential of iPSCs into a distinct functional “organ” (Ueda et al., 2010). Such progress including identification of Lgr5 as the intestinal stem cell marker and necessary growth factors has paved the way for methods of generating primary intestinal epithelial cultures, so-called mini-intestines. As a new paradigm for patient-relevant intestinal research in human intestinal physiology and pathophysiology, enteroids/colonoids developed from primary cultures of isolated intestinal crypts or stem cells, and intestinal organoids from iPSCs, are already being applied (Zachos et al., 2016). Intestinal organoids derived from iPSCs have resulted in the generation of more complex intestinal epithelium containing functional enterocytes, goblet cells, Paneth cells and endocrine cells (Miura and Suzuki, 2018).

Human intestinal and colonic epithelial organoids can be generated from adult normal tissues under specific 3D culture conditions containing EGF, noggin, R-spondin1, Wnt3a, nicotinamide, gastrin, A83-01 (an inhibitor of ALK5), and SB202190 (an inhibitor of p38 mitogen-activated protein kinase) (Sato et al., 2011). In addition, human colonic stem cells can expand and form organoids by 3D culturing with EGF, noggin, R-spondin1, Wnt3a, nicotinamide, gastrin, LY2157299 (an inhibitor of transforming growth factor beta receptor type 1 kinase), SB202190, and prostaglandin E2 (PGE-2) (Jung et al., 2011). Organoids with a defined foregut/gastric specification have been used to study disease such as *Helicobacter pylori* infection (McCracken et al., 2014). The complexity was increased by incorporation of primary stem cell-derived neural precursor cells in intestinal organoids, forming a functional enteric nervous system (Workman et al., 2017). One study has applied iPSCs with an intestinal phenotype on a microfluidic device, where the iPSCs were cultured within a gel (OrganoPlate) and differentiated step-wise within the plate. The cells formed an organoid tubular structure, lost their stem cell markers, and expressed mature markers for Paneth cells, enterocytes, and endocrine cells. The tubes had barrier-like properties shown by TEER and responded to pro-inflammatory cytokines (Naumovska et al., 2020).

Spence and colleagues published a method for generation of human intestinal organoids in a pro-intestinal culture system from embryonic stem cells and iPSCs by gradually adding essential factors to the culture medium in temporal series, mimicking intestinal development (Spence et al., 2011). These steps comprised, among others, endoderm formation from activin, posterior endoderm development from FGF/Wnt, and hindgut specialization. This method resulted in a 3D intestinal organoid consisting of a polarized, columnar epithelium in a villus-like structure, and crypt-like proliferative areas with intestinal stem cell markers. The epithelium contained functional enterocytes, as well as goblet, Paneth and enteroendocrine cells. In addition, the human intestinal organoids also contained mesenchymal cells, including fibroblasts and smooth muscle cells (Spence et al., 2011). Another study described the differentiation of healthy control human iPSCs into intestinal organoids *via* the addition of specific cytokine combinations to the culture medium before embedment into a basement membrane matrix-based pro-intestinal culture system (Lees et al., 2019). After being embedded, the organoids were supplemented with Noggin, R-spondin-1, epidermal growth factor (EGF), CHIR99021, prostaglandin E2, and Y-27632 dihydrochloride monohydrate. Manual disruption of the ultrastructure induced the formation of budding with crypt/villus structure. The differentiation within the organoids of goblet cells, enteroendocrine cells, Paneth cells, and polarized enterocytes, were confirmed by immunostaining of each cell subset, transmission electron microscopy, and quantitative PCR. This model was applied in modeling *Salmonella* infection by microinjection of the bacterium into the organoid lumen (Lees et al., 2019). Other available protocols exist, such as this more recent protocol, where Yamada and colleagues describe a protocol for generation of human iPSC-derived organoids, mono-layer formation, and their use in the evaluation of intestinal barrier functions. Here, the iPSCs were similarly subjected to sequential treatments with different cytokines and compounds and demonstrated as a physiologically relevant human platform tool for evaluating intestinal barrier integrity (Yamada and Kanda, 2021).

Other methods for generation of intestinal organoids, such as direct reprogramming technology of differentiated somatic cells also exist (Miura and Suzuki, 2018). Human intestinal organoids still have some drawbacks such as immaturity and lack of specific features of the adult intestine in absence of long-term culturing (Finkbeiner et al., 2015), but these studies show that great effort is being given to the development of protocols to approach *in vivo* conditions and multi-cellularity. A selection of available protocols for the generation of gut barriers using iPSCs with or without the presence of other cells is provided in Table 1.

**TABLE 1:** Overview of a selection of protocols for the generation of gut barriers using induced pluripotent stem cells with or without the presence of other cells.

Selected protocols for the generation of gut barriers					
Cells	Cell type	Protocol	Barrier tightness	Membrane	Reference
Human	Intestinal epithelium	1. Endoderm differentiation: 3 days, added GDF8, GSK3b inhibitor + B27	937 Ω x cm^2^ after 31 days culture	Transwell inserts coated with Geltrex	Kauffman et al. (2013)
A1145A		2. Hindgut differentiation: 7 days, add KGF + RA			
B2198A		3. Intestinal differentiation: >26 days, EGF, Noggin, R-Spondin 1			
C2198A					
C2200B (source: Johnson & Johnson)					
Human	Enterocyte-like cells	1. Endoderm differentiation: 4 days, Activin A+ overexpression using FOXA2	420 Ω x cm^2^	Matrigel Matrix Growth Factor Reduced–coated BD Falcon cell culture inserts	Takayama et al. (2019)
YOW-iPSCs		2. Intestinal progenitors: 15 days, BIO + DAPT + overexpression using CDX2			
		3. Enterocyte-like cells: 15 days, BIO, DAPT, EGF, SB431542, Wnt-3A			
iPSCs generated in-house	Enterocyte-like cells	1. Endoderm differentiation: 3 days, Activin A+ FBS	238 Ω x cm^2^	Transwell inserts coated with Matrigel + Y-27632	Kwon et al. (2021)
		2. Hindgut differentiation: 4 days, FGF4, CHIR99021, FBS			
		3. Enterocyte progenitors: 7 days (up to 10 passages) DMEM/F12, EGF, R-spondin1, insulin, FBS, B27, NEAA			
		4. Mature enterocytes: 10–14 days, seeded on membrane, Y-27632, DMEM/F12, EGF, Wnt-C59, VPA acid, FBS, B27, N2, NEAA			
Mouse 20D-17 (Riken BRC, Tsukuba, Japan)	iGUT organoid	1. Embryoid Body: 6 days, hanging drops, absence of LIF	Not measured	None	Ueda et al. (2010)
		2. iGUT: 21 days plated on gelatin			
Human iPSC lines (3.5, 3.6, 16.5)	Intestinal organoids containing progenitors, enterocytes, Paneth cells, myoepithelial cells, smooth muscle cells	1. Endoderm differentiation: 3days, Activin A, nodal-related TGFb molecule	Not measured	None	Spence et al. (2011)
		2. Hindgut differentiation: 4 days, Wnt3a, FGF4			
		3. Spheroids: 21 to >100 days, embedded in Matrigel, R-Spondin1, Noggin, EGF, then N2, B27			
Human 253G1 (Riken BRC, Tsukuba, Japan)	Intestinal organoids	1. Endoderm differentiation: 1day, B27, Activin A, CHIR99021, 2days, B27, Activin A	Without RA 68.5 Ω x cm^2^	None	Yamada and Kanda (2019)
		2. Hindgut differentiation: 4 days, B27, CHIR99021, Activin A, FGF4	With RA 97.9 Ω x cm^2^		
		3. Spheroids: 14 days, embedded in Matrigel, B27, N2, EGF, Noggin, R-spondin 1			
		4. Plated spheroids: 4 days, plated on Type1 collagen, B27, N2, EGF, Noggin, R-spondin1, HGF, Wnt3a, SB202190, A83-01, BSA, Y-27632 ± RA 2 days			

#### 2.1.2 Studying the leaky gut using iPSCs

The gut barrier can be compromised due to different factors, such as stress, diet, genetics, cancer, dysbiosis, and other related mechanisms (Vancamelbeke and Vermeire, 2017). In the healthy gut, the barrier function of the epithelium is maintained by the tight junctions. In pathological conditions where the tight junctions are disrupted, increased amounts of potentially pathogenic ligands leak through the barrier to the basolateral receptors. In turn, TLR expression is upregulated and positions of the TLRs are often altered in pathological gut environments, leading to increased inflammatory responses, as have been shown in Crohn’s disease patients where the basolateral expression of TLR4 in the colon is shifted to an apical location (Cario and Podolsky, 2000). A leaky gut barrier has been associated with systemic inflammation affecting the metabolic and immunological state of the body, the so-called leaky gut syndrome. A compromised barrier allow passage of toxins, antigens, bacteria, and environmental factors from the lumen to enter the blood, which may trigger the initiation and development of autoimmune disease in sensitive individuals for instance with a genetic predisposition (Mu et al., 2017). A range of studies have applied iPSC-derived intestinal barriers and organoids in studies of impaired gut functions. Intestinal organoid cultures are suitable as a model system for studies of the pathways and mechanisms involved in epithelial damage and repair (Blutt et al., 2019). Blutt and colleagues highlight the usefulness of organoids for studying and defining regenerative pathways induced by radiation or chemical damage or from infectious insults to the epithelium, pathways which could be targets of preventive or therapeutic studies (Blutt et al., 2019). A model of intestinal permeability using iPSC-derived human intestinal organoids and human colonic organoids was used for studying barrier dysfunction in Inflammatory Bowel Disease (IBD) (Gleeson et al., 2020). The iPSCs were developed from healthy humans and from adult and early onset IBD patients, and cultured in a Transwell® system. Barrier integrity studies were carried out in the presence or absence of the pro-inflammatory cytokines, TNFα and IFNγ. Tight junction and adherens junction protein expression and location was evaluated by Quantitative real-time PCR, transmission electron microscopy (TEM), and immunofluorescence. Reported results were increased permeability after differentiation, and mislocalized E-cadherin and ZO-1 in TNFα and IFNγ challenged organoids with a corresponding decrease in mRNA expression. The iPSC-derived human intestinal organoids and human colonic organoids were both reported as physiological models with relevant responses to study barrier dysfunction of IBD (Gleeson et al., 2020).

Other aspects of epithelial functions have also been investigated using both iPSCs and embryonic stem cells. A study investigated chronic inflammation and fibrosis patterns of IBD by studying the timing of inflammatory and fibrotic responses during organoid development from the human embryonic stem cell line H1 (Kandilogiannakis et al., 2021). The expression of mesenchymal markers during their maturation process were studied along with effects of inflammatory stimuli on expression of fibrotic and immunological mediators. Epithelial tight junction components, CLDN1 and JAMA, responded to inflammatory stimulation independently of the culture passage. In contrast, the mesenchymal component of the organoids gradually declined through culture passages. This was shown by high expression of CD90, collagen type I, collagen type III, and fibronectin in early passages which gradually diminished in late passages. Hence, this model might be suitable for the study of epithelial-mesenchymal interactions in early passages (Kandilogiannakis et al., 2021). Chronic gut inflammation and visceral fat accumulation has been linked in Crohn’s disease, with the possible pathway of gut adipocytes triggering inflammation. In another study, intestinal epithelial cell monolayers from primary or induced pluripotent stem cell-derived intestinal organoids were produced, which were polarized and cytokine responsive (Takahashi et al., 2017). Upon co-culturing with differentiated adipocytes in a Transwell® system, pro-inflammatory genes were induced in both cell types despite the absence of immunocompetent cells, yielding a promising model of the intestinal epithelium-mesenteric fat signaling in Crohn’s disease and other obesity-related enteropathies (Takahashi et al., 2017).

Together these studies show that human iPSC-intestinal models can be used to study mechanisms and dysfunction of the gut barrier with potential use for personalized therapy (Lucafò et al., 2022), and with perspectives for applications in models of the GBA.

#### 2.1.3 Gut microbiota and intestinal cell models

A significant role in maintenance of a healthy gut barrier is played by the GM. Cell models of host–microbe interactions and innate immune functions is a step closer towards physiological relevance (Roh et al., 2021). Combining iPSC technology with GM-models will likely add significant knowledge on top of existing research from primary cells co-cultured with GM (Zhang et al., 2021).

A recent study examined different intestinal models and gut microbial metabolites in human iPSC-derived intestinal organoids, namely fetal-like intestine, intestinal stem cell-derived models, and intestinal disease models (Lee et al., 2022). The new isolated *Limosilactobacillus reuteri* strain DS0384 accelerated maturation of the fetal intestine using 3D human organoids with immature fetal characteristics. By metabolic profiling, they showed that the secreted metabolite N-carbamyl glutamic acid (NCG) was involved in beneficial effects of DS0384 bacteria-free supernatant applied to the intestinal maturation of organoids. This bacteria-free supernatant also promoted intestinal stem cell proliferation and was important for protection against cytokine-induced epithelial injury both in stem cell-derived and human iPSC-derived inflamed organoids (Lee et al., 2022). Forbester et al. applied human iPSC-derived organoids to investigate interactions with *Salmonella enterica* serovar Typhimurium. The study showed alterations in transcription, including cytokine patterns. After microinjection of the bacteria into the organoid lumen, the bacteria were capable of invading the epithelial barrier and residing in vacuoles (Forbester et al., 2015). These studies highlights the significance of GM in intestinal health as well as the usefulness of iPSC intestinal organoids in studies of probiotic applications and therapies for preventing gut barrier dysfunctions.

Developments have been achieved that can model the complex gut barrier-GM interface to some degree, comprising simulations of the microbial processes through the gastro-intestinal tract (Molly et al., 1993; Brück et al., 2003) and mucus-associated GM (Van den Abbeele et al., 2012). Host-responses are more difficult to address in an *in vitro* setting, and have primarily been studied using exposure of cultured cells to supernatants or short-term co-culture exposure in Transwell® systems (Parlesak et al., 2004) or mouse gut organoids (Lukovac et al., 2014). The advances in OoC have allowed for more complex GM-human cell co-culturing, although under aerobic conditions, which is a huge limitation for the physiological modeling of GM conditions. An advancement was presented with the host–microbiota interaction (HMI) module, which incorporated a semi-permeable membrane between co-cultures of human enterocytes and bacteria. The module includes a partitioning membrane, so that intestinal cells can be co-cultured with complex GM communities in microaerophilic conditions (Marzorati et al., 2014). Another step towards improvement was presented by Shah and colleagues, which developed a modular microfluidics-based human–microbial co-culture model (HuMiX) that enabled co-cultures of Caco-2 cells with facultative anaerobe and obligate anaerobe bacterial strains (Shah et al., 2016). The chip consisted of co-laminar microchannels, a medium perfusion microchamber, a human epithelial cell culture microchamber, and a microbial culture chamber. Each chamber with a separate inlet and outlet. Molecular analysis of the effect on the cell types were compared to published *in vitro* and *in vivo* data sets, showing similarity to the human physiological gut-microbe interface (Shah et al., 2016).

Caco-2 cells secrete distinct cytokines analogous to immune cells when they are challenged with different microbial stimuli. Consequently, they have been applied as a good model for studies of the specific immunological responses to different microorganisms and their products (Parlesak et al., 2004) and are still useful in more complex models as benchmark. A micro-fluidic intestine-on-a-chip system was presented in 2019 by Jalili-Firoozinezhad and colleagues (Jalili-Firoozinezhad et al., 2019). In this model, extended co-culturing of either Caco-2 cells or primary patient-derived organoids together with stable aerobic and anaerobic GM along a hypoxia gradient was possible, including control and real-time assessment of oxygen gradients of physiological relevance. Intestinal barrier functions and microbial diversity were sustained and consisted of >200 operational taxonomic units from 11 genera. Importantly, the ratios of Firmicutes and Bacteriodetes were similar to human fecal GM (Jalili-Firoozinezhad et al., 2019). Although the microfluidic OoC technology has greatly advanced the modeling of physiological-like GM-host interfaces, it seems that the use of iPSCs in these models is yet to be applied.

### 2.2 The blood-brain barrier

The brain’s vasculature forms the BBB, which is tighter than the vessel walls found in the systemic blood circulation and comprised by the multicellular neurovascular unit (NVU) (Zlokovic, 2011). It consists of microvascular endothelial cells, pericytes, microglia, and astrocytes (Figure 1). The BBB protects the brain tissues from potentially harmful circulating substances, while simultaneously allowing neurotransmitters, oxygen, metabolites, glucose, and other intrinsic essentials to pass through (Kadry et al., 2020). Passage across the selectively permeable barrier is possible by different active or passive transport mechanisms depending on molecular properties and barrier integrity (Obermeier et al., 2013; Ayloo and Gu, 2019). The specialized cell types of the NVU that forms the BBB along with the surrounding tissues and the frictional force of the blood stream (shear stress), regulates the complex permeable properties of the BBB (Cucullo et al., 2011). The brain microvascular endothelial cells constitutes the physical barrier with their intercellular tight junctions, formed by protein complexes including claudin and occludin, and adherens junctions, consisting of cadherin proteins (Bauer et al., 2014). Water-soluble elements are transported between the cells through the tight junctions, which block the passage of macro-molecules and importantly, restricts the diffusion of ions. The microvascular endothelial cells are highly polarized allowing for transcellular diffusion of lipid-soluble elements (Obermeier et al., 2013). Around 30% of these tightly adhered microvascular endothelial cells are encapsulated by pericytes, which are cells with stem cell like properties (Armulik et al., 2011). These cells secrete components of the extracellular matrix and guide astrocyte end-feet, affecting the basement membrane composition. The astrocytes are the governing functional cells of the BBB (Sofroniew and Vinters, 2010). They surround the microvascular endothelial cells and pericytes and reach the extracellular matrix with their end-feet (Wolburg et al., 2009). By secretion of various mediators through the end-feet, the astrocytes stimulate the other two cell types to regulate the BBB tightness, for instance through expression of tight junction proteins or regulation of blood flow (Abbott et al., 2010), and they seem to play a significant role in inflammatory conditions (Argaw et al., 2012).

#### 2.2.1 Modeling the blood-brain barrier using iPSCs

Since the emergence of the iPSC technology in 2006 (Takahashi and Yamanaka, 2006), numerous studies and reviews of iPSC-derived BBB models have been published (Lauschke et al., 2017; Raimondi I. et al., 2019; Fabre et al., 2019; Appelt-Menzel et al., 2020; Delsing et al., 2020; Workman and Svendsen, 2020; Wu et al., 2021). There has been a fast development from simpler BBB models using monocultures of brain microvascular endothelial cells, such as the immortalized human cerebral endothelial cell line hCMEC/D3, to subsequent developments of co-cultures with astrocytes, pericytes, and neurons (Lippmann et al., 2014; Yamamizu et al., 2017). These models encountered challenges with un-physiological TEER values and other factors depending on cell progenitor, species origin, and cell types (Lauschke et al., 2017; Raimondi I. et al., 2019). BBB organoids are now extensively applied as high-throughput screening models in drug discovery (Bergmann et al., 2018).

The engineering of microfluidic iPSC-derived OoC BBB models have greatly moved BBB studies forward. Several complex and dynamic chip models of the BBB are available with various designs according to the purpose of the model (barrier dysfunctions, drug delivery, etc.). Griep et al. constructed a model with two layers of poly-diemethylsiloxane/PDMS separated by a Transwell®-like membrane with included electrodes for TEER measurements (Griep et al., 2013). A more complex model was proposed by Booth and Kim with a multi-layered design with central perfusion channels and shear stress mimicry, albeit introducing critical assembly, accessibility, and seeding challenges (Booth and Kim, 2012). Brown and colleagues developed a similar complex model, using co-cultures of four cell populations (primary human brain-derived microvascular endothelial cells, primary astrocytes, pericytes, and iPSC-derived cortical glutamatergic neurons) with independent perfusion channels and embedding of neurons in a 3D collagen extracellular matrix-like gel (Brown et al., 2015). The importance of the extracellular-matrix has also been emphasized and models have been developed to incorporate this (Adriani et al., 2017; Campisi et al., 2018). Campisi et al. developed a perfusable 3D microvascular network BBB OoC model using a combination of cells from the NVU embedded in a fibrin gel (Campisi et al., 2018).

Vatine and colleagues composed a human OoC model of the BBB using iPSC-derived brain microvascular endothelial-like cells, astrocytes, and neurons (Vatine et al., 2019). The endothelial-like cells formed a tight monolayer expressing specific markers of the brain vasculature. The BBB chip exhibited physiologically relevant TEER values and predicted pharmacological blood-to-brain permeability. The researchers perfused the vascular lumen with whole blood and observed that the microengineered capillary wall protected neural cells from plasma-induced toxicity. In turn, by using patient-derived iPSCs from individuals with neurological diseases, the model could be used to predict a disease-specific lack of transporters and barrier integrity disruption. This model seems to recapitulate complex BBB functions and could be a future platform for modeling genetic neurological disorders, applied in drug screening and in personalized medicine (Vatine et al., 2019). Recent advances in OoC BBB models stem from the option of applying fluid flow (Workman and Svendsen, 2020) and thus enabling studies of how iPSCs-derived brain microvascular endothelial cells respond to flow-induced shear stress. It has been demonstrated in such a model, that the cells do not elongate or align with the flow direction, a unique property of brain endothelial cells (DeStefano et al., 2017). Instead, they respond dependent on force at the transcriptional level (Vatine et al., 2019). Another advantage of flow, is the possibility to add immunomodulatory signal molecules, such as pro-inflammatory cytokines, which was shown to upregulate endothelial adhesion molecules and increase adherence of perfused leukocytes (Linville et al., 2019). Progress has also been made by modifying extracellular matrix composition (Katt et al., 2018) and incorporating developmentally-inspired hypoxic conditions (Park et al., 2019). A selection of available protocols for the generation of BBB models using iPSCs with or without the presence of other cells is provided in Table 2.

**TABLE 2:** Overview of a selection of protocols for the generation of blood-brain barriers using induced pluripotent stem cells with or without the presence of other cells.

Selected protocols for the generation of blood-brain barriers					
Cells	Cell type	Protocol	Barrier tightness	Membrane	Reference
Mixed culture	Endothelial cells (iBMCS)	1. Endothelial differentiation: 6 days, -bFGF, 2 days hESFM medium (Life Technologies), bFGF, RA, platelet-poor bovine serum. Plated onto membrane in endothelial cell medium minus bFGF, minus RA	1500 Ω x cm^2^, 2 days post-seeding, remained above 1000 Ω x cm^2^ for 5 days	Porous flexible PDMS membrane coated with laminin on the brain side and a mixture of collagen IV and fibronectin for the endothelial cell side	Vatine et al. (2019)
Human	iPSC-derived neural cells	1. Neural progenitor (EZ) spheres: cultured in DMEM:F12 supplemented with B27, bFGF, EGF and heparin in ultra-low attachment flasks			
10 iPSC lines (Cedars-Sinai Medical centre)		2. Neural differentiation: seeded onto membrane and cultured with DMEM:F12, B27, N2, human BDNF			
Human BC1 GFP iPSCs	Brain microvascular Endothelial cells (dhRMECs)	1. Endothelial differentiation: 7 days, -bFGF, 3–4 days, endothelial cell medium (Life Technologies), bFGF, RA, platelet-poor human serum, plated on collagen IV and fibronectin and transferred to transwells	4,400 Ω x cm^2^, 48 h after seeding	Transwells coated with collagen IV and fibronectin	Katt et al. (2018)
Triple culture	Brain endothelial cells (iPSC-BMVECs)	1. Endothelial/Neural progenitor mixed cell differentiation: 6 days unconditioned medium, then in endothelial medium + RA coated with collagen, fibronectin	25,000 Ω x cm^2^ in pre-treated hypoxic conditions	Polyethylene terephthalate (PET) membrane coated on both sides with collagen IV and fibronectin	Park et al. (2019)
Human iPSCS IMR90-4 (WiCell Research Institute)		iPSC-derived endothelial cells cultured in hypoxic conditions 8 days prior to seeding			
Human primary astrocytes		Astrocytes and Pericytes seeded 7:3 ratio, cultured in astrocyte medium			
Human primary pericytes					
Human iPSCS BC1, iPS12 KW01, AD6	Brain endothelial cells (dhBMECs)	1. Endothelial differentiation: 6 days, unconditioned medium minus bFGF, + KSR, NEAA, 2–5 days, endothelial serum-free medium (Life Technologies) + human platelet poor derived serum + bFGF + RA	1330–2,260 Ω x cm^2^, 2 days after seeding	Cylindrical channel coated with type I collagen gels, cross-linked with genipin and coated with fibronectin and type IV collagen. ROCK inhibitor Y27632 added	Linville et al. (2019)

#### 2.2.2 Studying the leaky blood-brain barrier using iPSCs

A leaky BBB or barrier dysfunction is associated with many late-stage neurodegenerative diseases and has been proposed as a factor in a novel infectious/inflammatory etiology of Alzheimer’s (Rutsch et al., 2020; Goyal et al., 2021). The contributions of iPSC-derived BBB organoid models in the studies of neurodegenerative diseases, such as AD, PD, Huntingdon’s, and Amyotrophic Lateral Sclerosis, has been thoroughly reviewed elsewhere (Chang et al., 2020; Workman and Svendsen, 2020; de Rus Jacquet et al., 2021; Wu et al., 2021). Briefly, one of the major breakthroughs of the iPSC technology, is the possibility of applying patient- or disease-specific iPSCs in BBB models, making it possible to investigate intrinsic BBB perturbations. One study investigated whether selected mutations associated with neurodegenerative diseases contributed to BBB impairment in monolayers of human iPSC-derived brain microvascular endothelial cells from three healthy persons and eight patients with neurodegenerative disease. Protein and gene expression of BBB biomarkers, TEER, and permeability of different markers (Lucifer yellow, D-glucose, rhodamine 123, and others) were measured. The results suggested that mutations associated with neurodegenerative disease can independently induce BBB dysfunction, implying that the accumulation of defects in brain microvascular endothelial cells could lead to impairment of the BBB (Katt et al., 2019). A similar study relating to multiple sclerosis, investigated if intrinsic alterations in the BBB of patients contributed to pathogenesis. By using iPSCs from healthy controls and multiple sclerosis patients and differentiation to brain microvascular endothelial-like cells, the authors found that the patient-derived cells had impaired tight junction integrity, barrier properties, and efflux pump activity. In turn, the cells were found to have an inflammatory phenotype with increased expression of adhesion molecules and immune cell interactions, and some of these effects were countered with activation of Wnt/β-catenin signaling in patient-derived progenitor cells (Nishihara et al., 2022).

Advanced BBB models using iPSCs can accelerate the field of BBB-transport and -delivery of drugs. The majority of therapeutic agents do not cross the BBB. Transient BBB opening with the hyperosmotic agent mannitol was examined in a tissue-engineered microvessel model using stem cell-derived human brain microvascular endothelial cells perturbed with clinically relevant mannitol doses. By live-cell imaging, the study showed that mannitol caused dose-dependent and spatially heterogeneous increases in paracellular permeability by formation of transient focal leaks. The degree of BBB and recovery could be modulated by treatment with basic fibroblast growth factor. Hence, tissue-engineered BBB models may aid in mechanistic discoveries and improve therapies for treatment of CNS disease (Linville et al., 2020).

## 3 Modeling the gut-brain axis

Ideally, a complex *in vitro* model of the GBA should mimic neuronal, endocrine/hormonal, immunological, and microbe-derived communication pathways between realistic microenvironments of the gut barrier, BBB, and brain. As presented in previous sections, each component of such a model is complex by itself, both in regards to cell types and functions and in terms of the needed bio-ingenuity. The use of scaffolding has moved the 3D modeling field forward. For example, the human intestine has been mimicked using different approaches, including, a bioengineered 3D porous silk protein scaffold system with a hollow channel forming a more physiologically relevant representation of the residential microenvironment (Chen et al., 2015), a 3D-innervated tissue model (Manousiouthakis et al., 2019) and a 3D bioelectronic tubular electroactive scaffold model (Moysidou et al., 2021), as well as models which are able to mimic peristalsis (Kim et al., 2012). In turn, advancements in integration of sensing and detection mechanisms have further contributed to the possibilities (Signore et al., 2021). However, iPSCs-derived cells were, to the best of our knowledge, not applied in these models.

**FIGURE 2:**
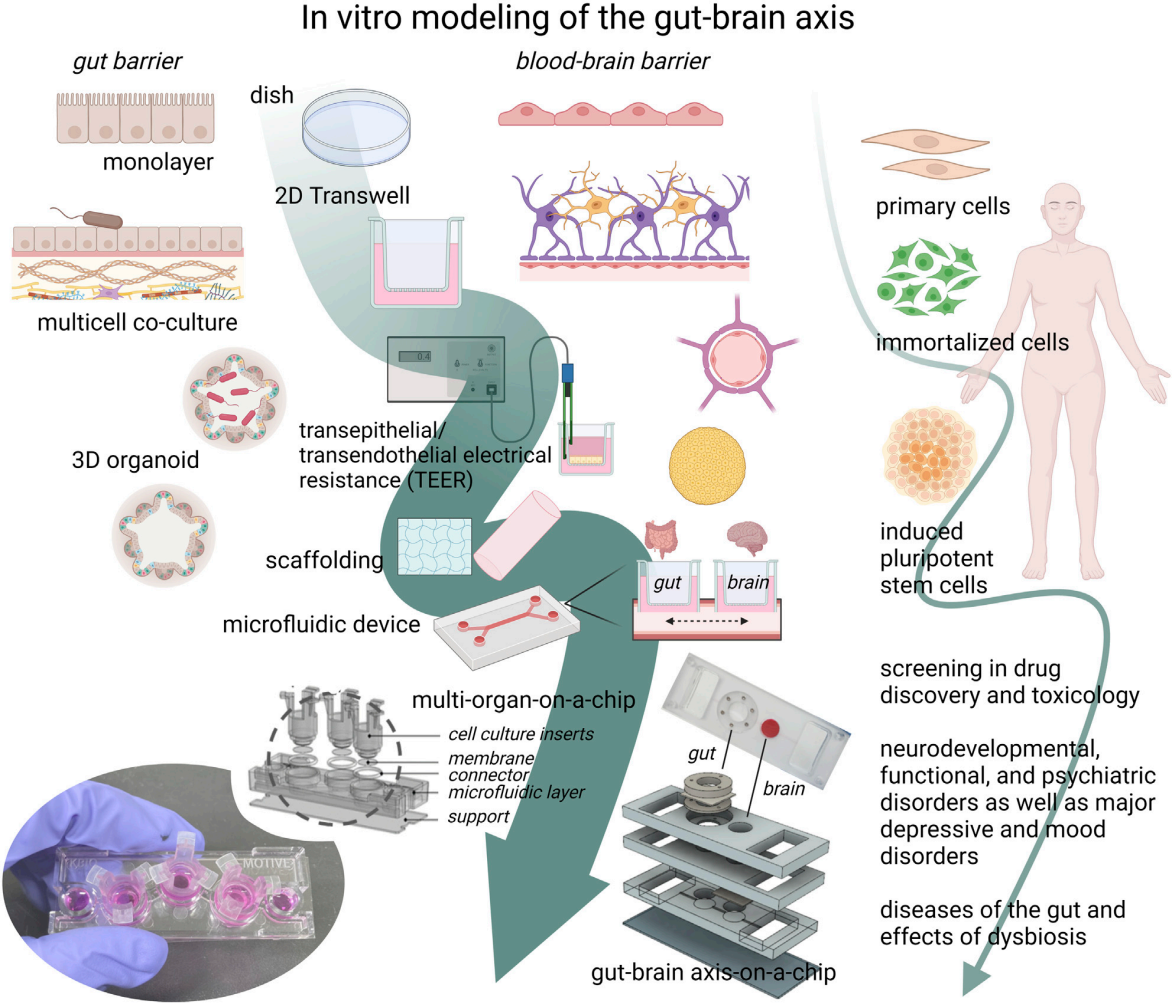
The development towards complex gut-brain axis models. The gut microbiome (GM), the gut barrier, and the blood-brain barrier (BBB) are key elements of the gut-brain axis (GBA). Individually, they have been extensively modeled with different *in vitro* approaches, ranging from mono- and multi-cellular co-cultures with or without gut microbe exposure, to 3D organoids or tissue models. Cell models can be derived from animal or human primary cells, developed from immortalized cell lines, or from stem cells, of which the induced pluripotent stem cell (iPSC) technology has advanced the cell model field forward. With this method, patient- or disease specific cells can be studied in an *in vitro* setting, providing a platform for drug screening in relevant complex human cell models. The advances in organ-on-a-chip technology and induced pluripotent stem cell (iPSCs) methods may together contribute further to the physiological complexity of gut-brain-axis-on-a-chip models, with which new mechanistic hypotheses and functions can be studied. The ability to mimic the complex physiological functions of the GBA *in vitro* is needed in basic research as well as in disease research of psychiatric, neurodevelopmental, functional, and neurodegenerative diseases, such as Alzheimer’s disease. (The figure was created with Biorender. GBA- and organ-on-a-chip figure and image were kindly supplied by Professor Jong Hwan Sung, Dep. Of Chemical Engineering, Hongik University, Seoul, Korea).

It is evident that in advanced models of the GBA, the role of the gut microbiome (GM) needs to be addressed and significant functions kept in mind, depending on the scope of the GBA model. GM-mediated functions and signaling relevant for the GBA are numerous. These may include neuronal pathways related to enteroendocrine sensing of metabolites, interoceptive signals, and homeostatic reflex loops to regulate GI motility, blood flow, and secretion (Porreca et al., 2002; Mayer and Tillisch, 2011; Carabotti et al., 2015; Mayer et al., 2022), and a direct synapse to vagal neurons through neuropod cells (Kaelberer et al., 2018). A relevant new research area within sensory neurobiology has emerged, namely the field of gut-brain sensory transduction of stimuli for the brain to guide behavior, such as exercise (Kaelberer et al., 2020; Dohnalová et al., 2022). The enteroendocrine cells of the gut produce >90% of the body’s total serotonin (5-hydroxytryptamine), which is an important regulator of secretion and motility of the gastro-intestinal tract (Kim and Camilleri, 2000). In turn, neuroendocrine hormones, dopamine and norepinephrine, which are released during stress *via* the hypothalamic-pituitary-adrenal axis, influence GM composition and gut barrier permeability (de Punder and Pruimboom, 2015). Soluble biochemical factors released by the GM, called the secretome, are important pathway constituents between gut and brain (Vancamelbeke and Vermeire, 2017; Raimondi I. et al., 2019). These include a wide range of GM-produced neuromodulators, such as serotonin (Enteroccoccus, *Escherichia* etc.) (Yano et al., 2015), γ-aminobutyric/GABA (Bifidobacterium, *Lactobacillus*) (Strandwitz et al., 2019), and acethylcholine (*Lactobacillus*) (Nimgampalle and Kuna, 2017). The molecular functions of the GM can be classified into host-derived metabolites, such as bile acids and steroid hormones, or dietary metabolites (Needham et al., 2020). Examples of important GM functions are amino acid metabolism and fermentation of indigestible complex carbohydrate polysaccharides, producing short-chain fatty acids, which are important cellular energy sources and systemic signal molecules. Other metabolites are those of plant-derived polyphenols and the generation of vitamins, lipid metabolites, hydroxy fatty acids, and sphingolipids. In turn, microbial cell wall components, the microbe-associated molecular patterns (MAMPs), are important signal molecules of the GBA (Needham et al., 2020). The two main bacterial cell wall components peptidoglycan, present in most bacteria, and lipopolysaccharide (LPS) of Gram-bacteria are reportedly translocated and, for LPS, co-localized with the receptor in the brain, and have been shown to affect brain development and health. LPS is a well-known mediator in research to induce sickness behavior, acute depression, and promote and initiate inflammation in both *in vitro* and *in vivo* disease models (Hug et al., 2018; Needham et al., 2020). In turn, LPS has been found to increase the permeability of the BBB (Xaio et al., 2001). The short-chain fatty acids are important for normal gastro-intestinal barrier functions and host metabolism, and have been reported to contribute to neuro-immune regulation, by increasing the expression of tight junctions proteins in the BBB (Xiao et al., 2020). Importantly, the ongoing immune surveillance, signaling, and immune cell functions associated with the GM and the intestinal tract plays a key role in GBA-signaling (Agirman et al., 2021; Liu et al., 2021). Major questions regarding the GBA and which GM-host interactions are vital in a model still remains, including solutions to closer mimic *in vivo* conditions *in vitro* (Figure 2). Some unresolved matters of GM-gut barrier models pointed out by Roh et al. (2021), are: enabling longer co-culturing times mimicking chronic states, improvement of intestinal mucus secretion and thickness, incorporating innate intestinal immune functions, influence of flow perfusion on the epithelial cells, translational aspects of knowledge from animal models in drug discovery, and incorporation of the *in vivo* variance in dietary antigens, digestion, species, age, and gender effects in the models (Roh et al., 2021).

To study how microbiota changes affect the brain using GBA OoCs, researchers can choose to study pathological changes to the axis itself, the barriers, and/or the brain. If studying cultured brain cells/tissue, it would be important to study relevant phenotypes for the disorder or disease of interest. For example, plaques and tangles arise in the brain of AD patients (Duyckaerts et al., 2009), and α-synuclein aggregation develops in the brains of PD patients (Halliday and McCann, 2010). In the case of depression, this might be harder to measure *in vitro*, as cortisol levels rise and some regions of the brain shrink or expand. However, neuroinflammation is a feature (Han and Ham, 2021) that could be measured if microglia were added to the OoC models. In addition, neuroinflammation is also reported in AD and has already been implicated to be due to GM perturbations (Heneka et al., 2015; Chen et al., 2020; Rutsch et al., 2020; Goyal et al., 2021; Bäuerl et al., 2022; Sorboni et al., 2022). In the case of the gut barrier and BBB, there is evidence that these become dysregulated and may lead to disease onset. In order to model these barriers, it is important to understand the cellular complexity and ensure that phenotypes can be replicated similar to that observed in patients.

In a physiological relevant GBA model, the cross-barrier effects from one barrier to the other is a key physiological phenomenon to be able to study. This means that the systemic-like microfluidic accessible interface must readily connect both barriers uni- or bi-directionally, dependent on the hypothesis being investigated using the model. The poly-diemethylsiloxane/PDMS microphysiological microfluidic chip systems have greatly advanced chip technology (Raimondi I. et al., 2019) (Bhatia and Ingber, 2014). The microfluidic technology make it possible to include exposure to physical factors including flow and shear stress. This also improves the physiological exchange of nutrients and metabolites and the integration of sensors and electrodes facilitates direct measurements. In a larger multi-compartmental chip system, each tissue/cell- and fluid compartment must be individually established, maintained, assembled, sampled, and analyzed.

The integrity of the gut barrier and the BBB is determined by similar components, which can be assessed in experimental conditions by electron-microscopy imaging, gene expression of tight junction proteins, or measurement of the diffusion of tracer molecules or markers such as dextran, glucose, or insulin (Saunders et al., 2015; Sun et al., 2021). Measurement of the transepithelial/transendothelial electrical resistance (TEER) arising from the ion restrictive conditions is a key parameter of barrier tightness, and embedded microelectrodes for TEER measurement is a component of current microfluidic organ-on-a-chip models. Gut epithelial cells are classified based on their TEER values as “tight” (2000 Ω cm^2^), “intermediate” (300–400 Ω cm^2^), or “leaky” (50–100 Ω cm^2^). The TEER values of different cell lines in BBB cell models vary significantly dependent on cell type, co-culture or mono-culture, and species-origin (Srinivasan et al., 2015). A vertical modular GBA-on-a-chip was recently presented by Kim and colleagues (Kim et al., 2021). In this chip system, human gut epithelial (Caco-2) and human/murine brain cells (primary human brain microvascular endothelial cells and murine brain endothelial cell line (bEnd.3) were co-cultured forming a gut barrier and a BBB, respectively, connected by microfluidic channels. To simulate an inflammatory response of the GBA, LPS exposure was applied showing decreased TEER values and increased permeability of both barriers, although this depended on culture conditions. When exposing the cells to butyrate, the integrity of both barriers were improved (Kim et al., 2021). To our knowledge, this is currently the only published chip model of the GBA *per se*, incorporating both barriers with a fluidic system in between. However, this model might be developed further by incorporating more cell types and iPSC-derived cells. A chip model to study gut-liver-cerebral interactions was proposed and applied for studying PD, where microphysiological systems of primary human gut and liver were connected with a human iPSC-cerebral system and a common culture medium containing T-cells circulated between the compartments (Trapecar et al., 2021).

Large collaborative projects specifically aiming to develop GBA-on-a-chip models have moved the field forward. The MINERVA project ended in 2022 (ERC Grant agreement no.724734^1^) aimed at developing a microbiota-gut-brain multi-modular engineered platform to evaluate intestinal microflora impact on brain functionality (Raimondi I. et al., 2019; Raimondi M. T. et al., 2019). Important contributions came from this project, such as considerations of the role of GM in modeling the GBA (Ceppa et al., 2020) (Sardelli et al., 2021) and the development of an *in vitro* physiological brain-like hydrogel tissue model (Raimondi et al., 2020). The IMBIBE project (ERC Grant agreement no.723951^2^), ending in March 2023, aims to generate a complete platform of the human microbiota-gut-brain axis with integrated monitoring and sensing capabilities (Moysidou and Owens, 2021). Another large research platform is GUTVIBRATIONS (ERC Grant agreement no. DT-NMBP-23, under the Next-Generation Organ-On-Chip Call^3^), which aims at developing applicable gut modules and combining some of them into a next-generation gut-brain axis organ-on-chip.

## 4 Discussion

The two compartment barriers interconnected in the GBA, the gut barrier and the BBB, can be studied using NAMs, which enable us to model these microphysiological environments with various complexity. The gut barrier and the BBB are both multicellular in composition with essential histological structures and microenvironments not easily recapitulated *in vitro*. Most research has been carried out in monocultures and co-cultures, modeling a particular subsection of the GBA barriers. Despite these limitations, these *in vitro* models are invaluable screening tools in drug discovery and toxicology. Increasingly complex models have been proposed, such as incorporation of GM constituents in gut barrier models, and BBB models containing 3D extracellular matrix-like components. Whether to choose monoculture, co-cultures, or more complex 3D organoid models for a GBA-on-a-chip will depend on the research questions posed. In complex OoC models, considerations of seeding and culturing conditions of different cell types, accessibility, assembly, and other practical issues are complications, which may overshadow the benefits and if the research questions could be answered using simpler models.

There is great promise in using patient-derived iPSCs to build these models. However, there might be disease-related changes in the microphysiological environments that need to be incorporated to produce valid cellular models. For instance, the effects of GM-associated changes due to stress, diet, medicine, and environment in AD-patients might influence the immune environment and change gene signaling. Although the use of iPSC-derived cells from the same source is likely to reduce variation and mimic the inter-compartmental microenvironment of an individual’s GBA to a higher degree, major challenges remain regarding chip compartment design. The simplest method is to culture each cell type separately and then combine differentiated cells into one chip system. However, there might be missing developmental co-factors between cell types, which could be overcome by co-culturing instead. To be able to direct iPSCs towards a specific lineage, the needed factors have to be incorporated in the media for each cell line at different time points. Hence, the microfluidic system must be designed to allow differentiation events of media, while simultaneously being inter-compartmentally connected by fluids.

In summary, as for all models of biological systems, considerations of the research questions or suggested hypotheses must be aligned with the capabilities of the GBA model. Current protocols and technology have come far in adding complexity, but incorporation of bacterial, immune, and neuronal pathways in GBA models remains a challenge, as well as the optimal design of the chip itself. Although animal models have paved the way for the breakthroughs and progression in the understanding of the GBA, the fundamental questions of exactly when, how, and why still remain unanswered. The research of the complex GBA have relied on equally complex animal models. Instead, specific and concisely designed GBA-on-a-chip models might be better for solving specific questions. But there is still some work required to ensure these cell models can model the physiological conditions of the GBA. One key concept to deal with in animal models is variance, something that is mainly attempted to be reduced as much as possible, for instance by use of inbred strains and standardization of experimental conditions. Even so, we inherently accept that biological variance is an intrinsic prerequisite in animal models, and in some circumstances also considered positive as models of real life. Another key discussion point is translationability. Many years of research have gone by working with and around variance and translationability in animal research. This still needs to be addressed in organ-on-a-chip models, which are still in an early phase of development. Chips can be highly variable in designs and tissues, for instance with modules in vertical stacks or horizontal series. These design-specific possibilities and parameters should be taken into consideration, when working towards validating these chips across laboratories. There is likely not one chip to fit all research questions. One advantage of these chips is the ability to test reproducibility of results by using several chips seeded with the same parental cell line. Research using these chips remain limited to testing questions that affect cellular responses and genetic, cell functional changes. However, they allow for vigorous hypothesis generation and testing prior to validation in animal models, which is an approach that contributes to reduction of animal use. The applicability of these chips is wide, both within disease research, but also in toxicological studies of adverse outcome pathways.

A progressive shift in development of new methods for building chips and for producing tissues from iPSCs has allowed researchers to ask important scientific questions on the gut and brain using cell models rather than animals (Figure 2). However, the field is in its infancy in regards to applying iPSCs in the production of GBA chips. Societal awareness and pressure to reduce animal research has led to increased funding for the development of NAMs and an increased surge in research activities will help push the field forward. Interdisciplinary collaborations between bioengineers, bioinformaticians, biochemists, *in vivo* and *in vitro* scientists are necessary to overcome the complexity in modeling the GBA. The promise of iPSC technology and the perspectives of its use in GBA-on-a-chip models, will likely contribute to significant developments in the coming years.

## Footnotes

1
https://doi.org/10.3030/724734.

2
https://explore.openaire.eu/search/project?projectId=corda__h2020::e466943a864a1aac0737804fb7c634c3.

3
https://gutvibrations.org/gutvibrations/.

## Abbreviations

AD, Alzheimer’s disease; BBB, Blood-brain barrier; ENS, Enteric nerve system; FMT, Fecal microbiota transplant; GBA, (Microbiota-) gut-brain axis; GM, Gut microbiome; iPSCs, Induced pluripotent stem cells; NAMs, Non-animal models; NVU, Neurovascular unit; MAMPs, Microbe-associated molecular patterns; OoC, Organ-on-a-chip; PD, Parkinson’s disease; TEER, Transepithelial/transendothelial electrical resistance; TLRs, Toll-like receptors.
